# The Great Mimicker of Gastric Cancer: A Case Report of Ménétrier's Disease

**DOI:** 10.7759/cureus.55076

**Published:** 2024-02-27

**Authors:** Sarmista Roy, Sushanto Neogi, Anubhuti Chaturvedi, Reena Tomar

**Affiliations:** 1 Department of Surgery, Maulana Azad Medical College, New Delhi, IND; 2 Department of Pathology, Maulana Azad Medical College, New Delhi, IND

**Keywords:** upper gi endoscopy, gastric cancer surgery, gastric lymphoma, upper gi bleed, foveolar hyperplasia, protein losing enteropathy, proximal gastrectomy, hematemesis, hypertrophic gastropathy, ménétrier's disease

## Abstract

This is the case of a 52-year-old Indian lady who presented with hematemesis, severe anemia, and an abdominal lump in cardiac failure. On radiographic evaluation, the lesion appeared to be gross circumferential asymmetric proximal gastric wall thickening, with suspicion of gastric lymphoma or tubercular hypertrophic gastritis. After stabilization with multiple transfusions, she underwent proximal D2 gastrectomy with esophago-gastric anastomosis and a total splenectomy.

Grossly, the gastric rugae appeared to be hypertrophied and firm. No growth was identified grossly; however, necrotic areas were identified at the distal end. Microscopic examination of multiple sections studied showed significant foveolar hyperplasia, tortuous glands, and a few cystically dilated foveolar glands, which were limited up to the muscle layer. Mild serosal congestion was seen. No atypia or invasion was seen. An impression to consider is the possibility of Ménétrier’s disease (MD).

MD is an acquired protein-losing enteropathy with giant gastric rugal folds, decreased acid secretion, and increased gastric mucous production. Radiographically, endoscopically, and grossly, the condition can be confused with malignant lymphoma or carcinoma. It is difficult to diagnose, and histopathological confirmation of the resected specimen is needed for a definitive diagnosis.

Our intention in presenting this case is to emphasize that MD can present as massive hematemesis and should be considered in a differential diagnosis. Surgical treatment by total or partial gastrectomy is recommended for cases with persistent, debilitating symptoms or a risk of cancer.

## Introduction

Ménétrier's disease (MD) is a rare, idiopathic, acquired premalignant disorder of the stomach with massive mucosal folds. It was first described by a French pathologist, Dr. Pierre Ménétrier, in an autopsy in 1888 [1-2]. The disease is rare, and as differential diagnosis is difficult on a clinical basis, it needs to be distinguished from the mucosal hypertrophy of Zollinger-Ellison syndrome, gastric carcinoma, and gastric lymphoma [2].

## Case presentation

A 52-year-old lady presented to the surgery OPD, Lok Nayak Hospital of Maulana Azad Medical College, New Delhi, with abdominal pain involving the epigastric and left hypochondriacal regions, which was a dull aching type, aggravated by food intake, and associated with non-bilious vomiting, hematemesis, and melena for the past 18 months. She also had abdominal distension, generalized swelling of the body, easy fatiguability, early satiety, reduced appetite, and a significant loss of weight for the past six months.

On examination, she had severe pallor, anasarca, facial puffiness, and a 10 x 7 cm palpable lump involving the epigastric, umbilical, and left hypochondriac regions, which were firm in consistency and moved with respiration; fingers could not be insinuated in the subcostal region. On chest auscultation, she had tachycardia with an ejection systolic murmur.

Routine blood investigations revealed a hemoglobin of 1.5 gm% with a peripheral smear examination showing a microcytic hypochromic picture, along with hypoproteinemia of 3.7 g/dl and hypoalbuminemia of 1.9 mg/dl. Other blood investigations were unremarkable.

On ultrasonography, the lump was identified as extensive diffuse thickening of the stomach wall, with a maximum thickness of 2.7 cm, showing increased internal vascularity with both arteries and venous blood flow, raising suspicion of neoplastic etiology of the stomach.

**Figure 1:**
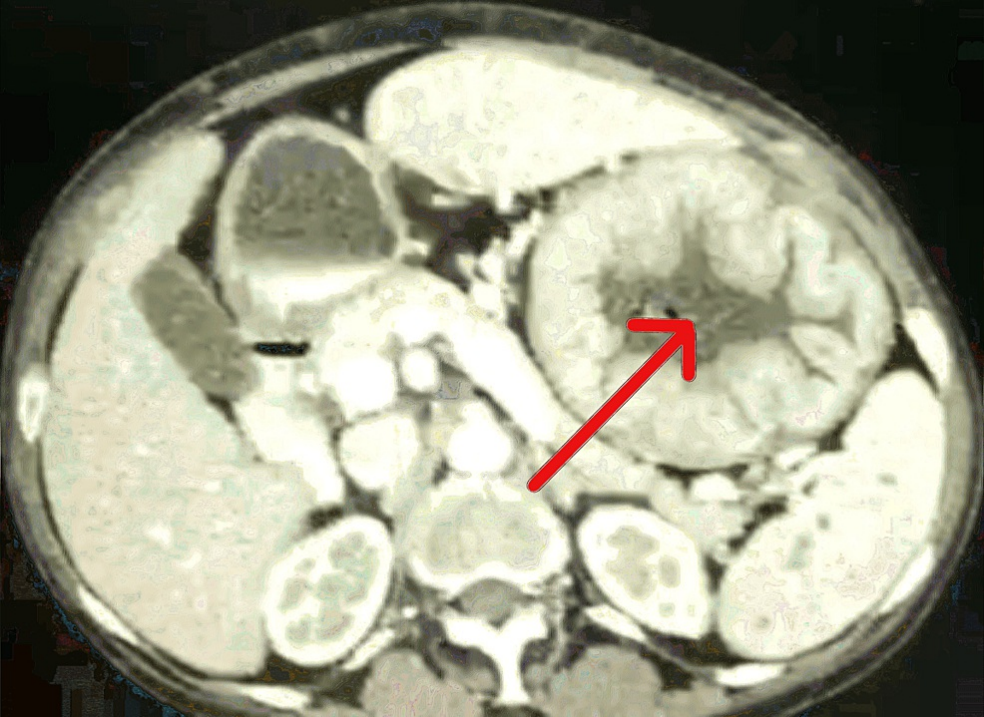
Contrast-enhanced CT of the abdomen: aneurysmal dilatation and circumferential asymmetric thickening of the stomach CT: computed tomography

On contrast-enhanced CT of the abdomen, as shown in Figure 1, there was gross circumferential asymmetric gastric wall thickening involving the cardia fundus and body (lesser curvature > greater curvature) sparing the pylorus, showing homogenous enhancement with aneurysmal dilatation of the stomach and passage of contrast distally. Adjacent fat planes were maintained, and multiple small perigastric and celiac lymph nodes were present. Diffusely scattered, multiple non-enhancing nodules were present in the spleen. We had a probable diagnosis of gastric lymphoma along with lymphomatous involvement of the spleen or tubercular hypertrophic gastritis with multiple splenic tubercular granulomas.

The upper gastrointestinal (GI) endoscopy revealed proliferative growth in the stomach that was extending into the fundus and proximal body; the scope is negotiable. The body revealed a few erosions and extensive growth, sparing the antrum and pylorus. Multiple biopsies were taken, and histopathology showed benign follicular hyperplasia.

The patient was taken up for surgery in view of clinical and radiological suspicion of malignancy/lymphoma, even though the endoscopic biopsy was suggestive of a benign lesion, as the patient had multiple episodes of hematemesis and severe anemia in cardiac failure. The patient underwent an open proximal D2 gastrectomy with esophago-gastric anastomosis and a total splenectomy.

**Figure 2:**
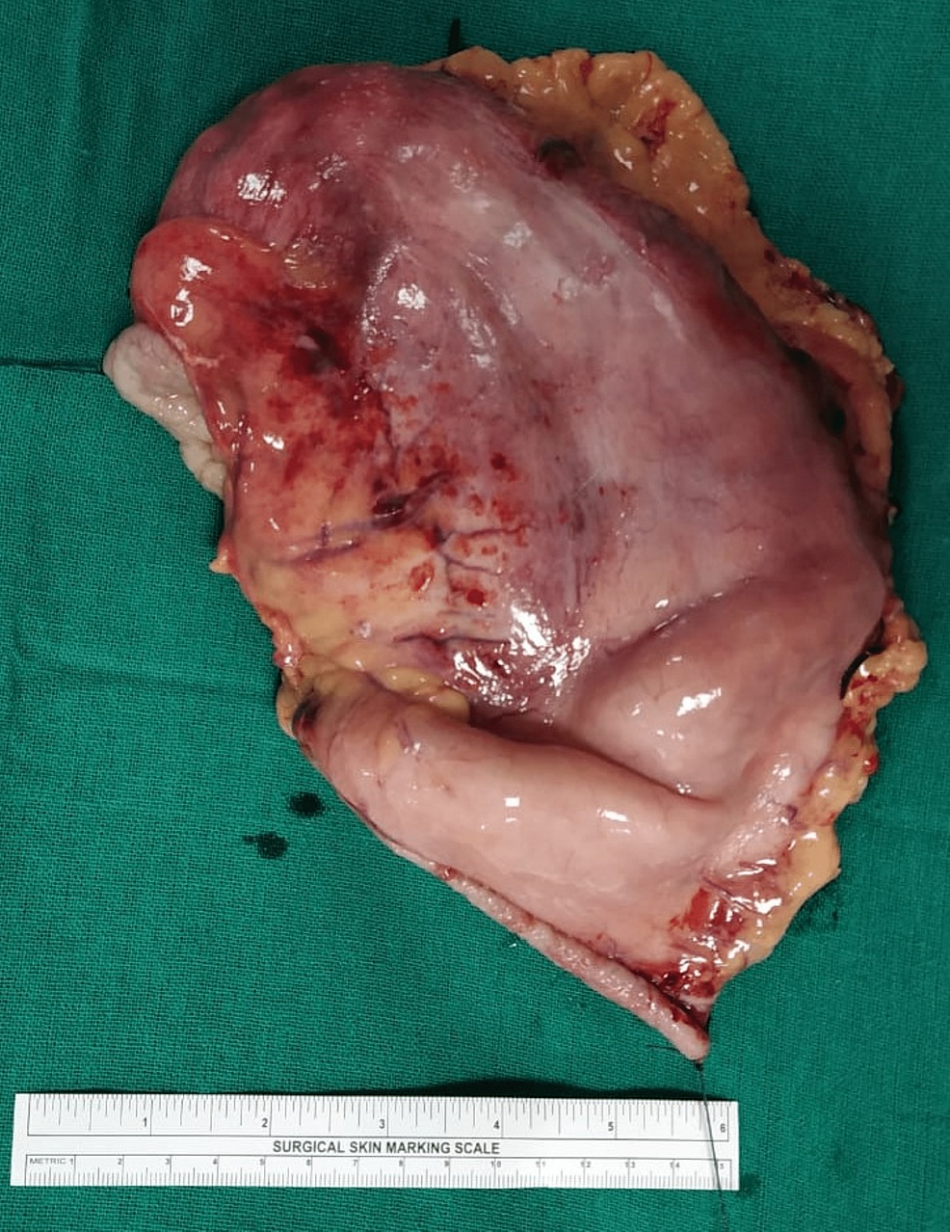
Gastrectomy specimen

Intraoperatively, there was circumferential growth from the gastroesophageal junction to the antrum with a thickened stomach (hyperplastic rugae); grossly, the spleen was normal. Figure 2 shows the gastrectomy specimen, which was 15 x 13 x 10 cm in size, grossly.

**Figure 3:**
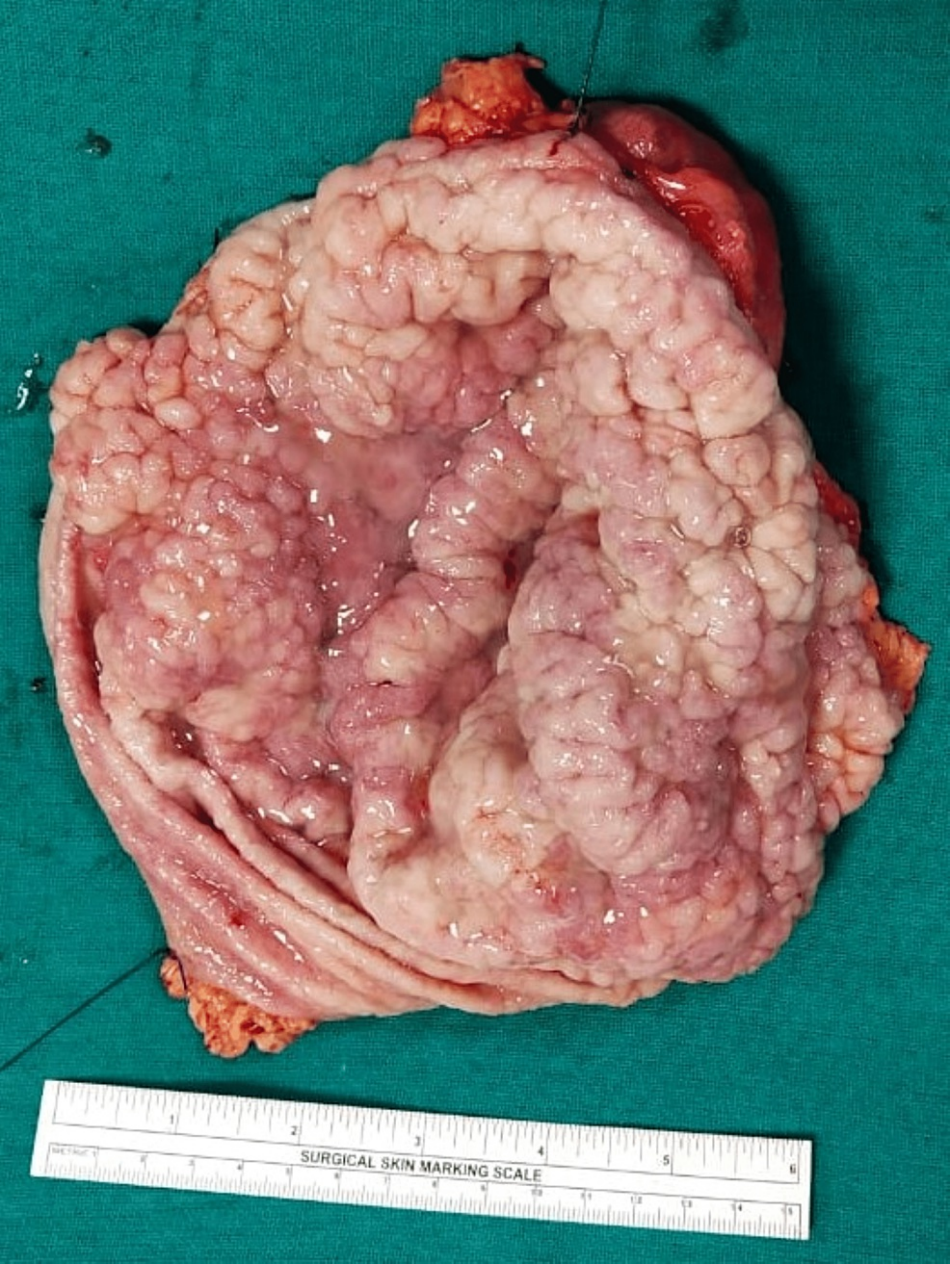
Cut-open gastrectomy specimen showing hyperplastic rugal folds

**Figure 4:**
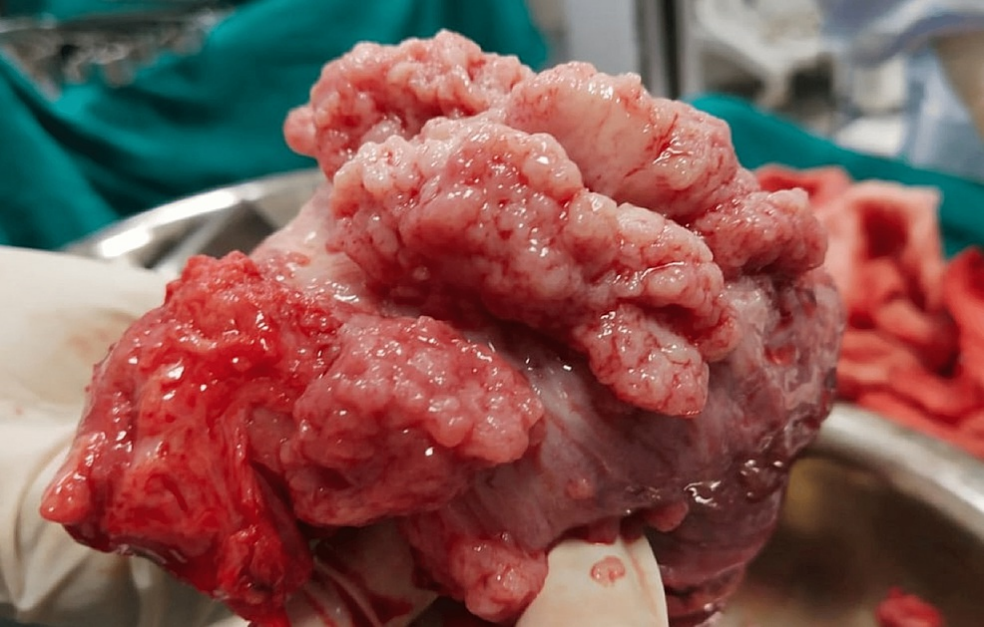
A closer view of the hyperplastic mucosal folds covered in mucous

On cutting open the specimen, as shown in Figure 3 and Figure 4, the gastric rugae appeared to be hypertrophied and firm. No growth was identified grossly; however, necrotic areas were identified at the distal end. The attached omentum measured 5 x 3 x 2 cm. No lymph nodes were identified.

**Figure 5:**
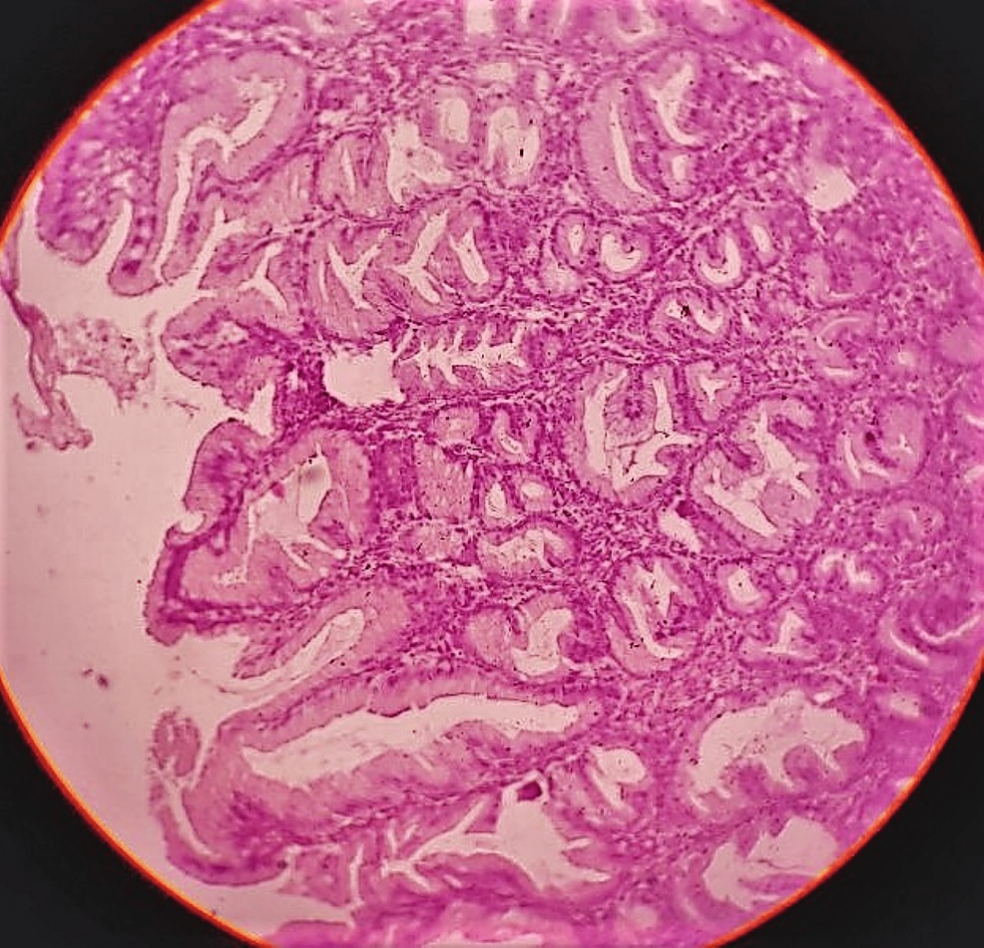
A 10x hematoxylin and eosin-stained slide showing foveolar hyperplasia, tortuous glands, and a few cystically dilated foveolar glands

**Figure 6:**
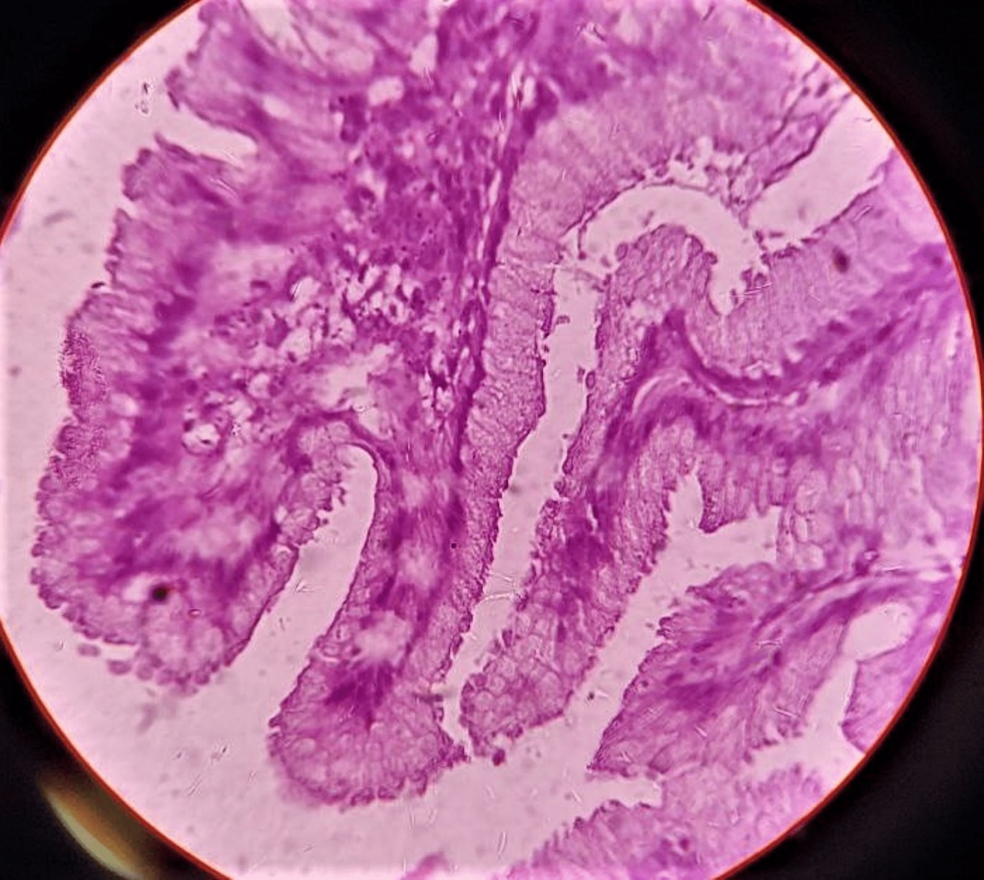
A 40x hematoxylin and eosin-stained slide showing foveolar hyperplasia

As seen in Figure 5 and Figure 6, microscopic examination of multiple sections showed significant foveolar hyperplasia, tortuous glands, and a few cystically dilated foveolar glands, which were limited up to the muscle layer. Mild serosal congestion was seen. No atypia or invasion was seen. An impression of hypertrophic gastropathy was made, and the patient was diagnosed with MD.

The splenectomy specimen grossly measured 10 x 7 x 4 cm, of which the outer surface was unremarkable. Microscopic sections revealed splenic congestion and the presence of seven lymph nodes, all of which showed reactive lymphoid hyperplasia. The patient was discharged on postoperative Day 13, and the stay was uneventful.

## Discussion

MD, being a rare disease, was very difficult to diagnose and needed the surgical specimen to come to a definitive diagnosis in this case. The usual symptoms are epigastric pain (65%), fatigue (60%), anorexia (45%), weight loss (45%), vomiting (38%), and edema (38%) due to the protein loss, but our patient presented an upper GI bleed that led to severe anemia and congestive heart failure, which is a rare complication [1-6]. A very rare presentation of this disease is gastroduodenal intussusception [7].

On blood investigations, there will be anemia with or without eosinophilia, lower serum albumin levels, normal to slightly high serum gastrin levels, and patients might be positive for *Helicobacter pylori *(*H. pylori*)* *and cytomegalovirus serology [1,5,8]. Our patient had anemia, hypoproteinemia, and hypoalbuminemia, but we did not get serum gastric levels, *H. pylori*, and cytomegalovirus cytology, as we were not suspecting MD at that point. MD patients have an increased gastric pH in most cases [1]. Radiologically, it shows a thickened gastric wall on a CT scan, as reported in our patient.

On endoscopy, MD shows enlarged gastric folds usually spare antrum, surface erosions, and copious amounts of thick mucus may form bridges across the gastric lumen and might obscure visualization [1,3]. Bjork et al. defined an enlarged gastric fold as measuring > 1 cm by radiology and that persists after air insufflation on endoscopy [9]. A full-thickness biopsy of the enlarged gastric folds taken using deep snares essentially shows a thick layer of adherent mucin, which usually survives processing. They retain mucosal architecture with gastric glands mostly found in parallel and with some areas of deep lateral branching and cystic dilatation, foveolar hyperplasia, lamina propria smooth muscle hyperplasia, and edema, with half the cases showing prominent eosinophils or plasma cell clusters in the lamina propria and a reversal gastric pit-to-gamma ratio [1,10].

Our patient's MD was a rare diagnosis; even experienced gastroenterologists did not suspect it from endoscopy, and even a biopsy was inconclusive. The surface mucous cells stain positive with PAS, MUC5AC, gastrokine, and TFF1. On immunohistochemical staining, there will be decreased parietal cells, decreased chief cells, and decreased expression of H+/K+ ATPase and pepsinogen. The cells will also be positive for Ki67 in the progenitor zone [11].

Two variants of MD have been defined histologically, i.e., hypertrophic lymphocytic gastroenteritis and massive foveolar hyperplasia, as compiled in Table 1 [1,8].

**Table 1:** Histological variants of MD MD: Ménétrier’s disease

Hypertrophic lymphocytic gastritis	Massive foveolar hyperplasia
Intraepithelial lymphocytes with increased inflammatory changes in lamina propria	No prominent inflammatory changes with rare intraepithelial lymphocytes
Gastric pit-to-gastrin ratio maximum of 3:1	Gastric pit-to-gastrin ratio maximum of >3:1

The assumed theory of MD is local overexpression of transforming growth factor alpha, which activates the epidermal growth factor receptors, which are a type of tyrosine kinase receptor that leads to selective expansion of surface mucous cells in the gastric body and fundus, leading to increased mucous secreting cells and thus mucus secretion, loss of protein, and a decrease in parietal cells, leading to less gastric acid [12].

Some cases are associated with *H. pylori* infection, and in children, it was associated with cytomegalovirus infection and showed spontaneous resolution with medical treatment with specific antimicrobial agents like the triple regimen and ganciclovir, respectively. The exact mechanism of viral infection in the pathogenesis of this disease is yet to be discovered [13-14].

The conservative management includes a high-protein diet, proton pump inhibitors, and the replacement of micronutrients [8]. A definitive treatment in view of future cancer risk and debilitating morbidity is surgical, subtotal, or total gastrectomy [2,15]. Octreotide at a dose of 100 to 600 micrograms daily has been shown to be beneficial in case reports as it acts by modulating the TGF-alpha-EGFR pathway, which is the underlying pathogenic mechanism [16]. In recent cases, cetuximab and monoclonal antibodies to EGFR have been successfully used to treat MD [11].

## Conclusions

MD is a rare diagnosis because of the lower incidence, especially if presented as hematemesis or an abdominal lump mimicking malignancy. We opted for surgical treatment in this case due to the higher suspicion of malignancy and the disease-causing debilitating effect on the patient. We recommend surgical treatment by total or partial gastrectomy with lymph node dissection in view of the high malignant risk and in cases presenting with severe symptoms. Further studies into the disease are required, and awareness among endoscopists about the appearance can help in early diagnosis and medical therapy.
